# Extracellular Vesicle: An Emerging Mediator of Intercellular Crosstalk in Lung Inflammation and Injury

**DOI:** 10.3389/fimmu.2018.00924

**Published:** 2018-04-30

**Authors:** Heedoo Lee, Eric Abston, Duo Zhang, Ashish Rai, Yang Jin

**Affiliations:** ^1^Division of Pulmonary and Critical Care Medicine, Department of Medicine, Boston University Medical Campus, Boston, MA, United States; ^2^Department of Internal Medicine, North Shore Medical Center, Boston, MA, United States

**Keywords:** macrophage-epithelium crosstalk, lung injury and inflammation, extracellular vesicles, exosome, microvesicle, apoptosis, apoptotic bodies, microRNA

## Abstract

Inflammatory lung responses are one of the characterized features in the pathogenesis of many lung diseases, including acute respiratory distress syndrome (ARDS) and chronic obstructive pulmonary disease (COPD). Alveolar macrophages (AMs) and alveolar epithelial cells are the first line of host defense and innate immunity. Due to their central roles in both the initiation and resolution of inflammatory lung responses, AMs constantly communicate with other lung cells, including the alveolar epithelial cells. In the past, emerging evidence suggests that extracellular vesicles play an essential role in cell–cell crosstalk. In this review, we will discuss the recent findings on the intercellular communications between lung epithelial cells and alveolar macrophages, *via* EV-mediated signal transfer.

## Introduction

Acute respiratory distress syndrome (ARDS) and acute lung injury (ALI) is fundamentally a syndrome characterized with an intense inflammatory response, severe injury to the epithelial/endothelial barrier, and alveolar edema (1–3). Overwhelming inflammatory responses cause collateral damage in lung tissue irrespective of the initial cause, patients with ARDS universally have high levels of inflammation and circulating cytokines. However, clinical trials using anti-inflammatory agents, such as glucocorticoids to treat ARDS have failed to improve outcomes (4). Chronic obstructive pulmonary disease (COPD) is also characterized by a heterogeneous lung inflammation (5) involving epithelial cells, alveolar macrophages (AMs), neutrophils, and T cells (6). To date, the knowledge on how pulmonary cells communicate with each other and subsequently trigger an inflammatory cascade remains incompletely understood.

Alveolar macrophages are a distinct resident population that comprises the majority of inflammatory cells in the healthy lung. They form the first line of host defense against inhaled dust and/or infection, working as antigen-presenting cells and releasing powerful pro-inflammatory cytokines to drive the inflammatory response required to fight infection. Macrophages are capable to directing the type and severity of inflammatory response based on the type of injury to the lung, and also plays an important role in the resolution of inflammation and lung injury. Due to its central roles in both the initiation and resolution of inflammatory lung responses, AMs constantly communicate with other lung cells. The interactions between macrophage–neutrophil, macrophage-recruited macrophages, macrophage-lymphocyte, and macrophage-mesenchymal stem cell have been well described previously (7–10). In this review, we will discuss a novel paradigm on how macrophage–epithelial cell crosstalk occurs *via* extracellular vesicles (EVs) and EV-containing microRNAs (miRNAs).

The alveolar epithelium, with their large surface area acts as the first-line defense against insult, and contribute to the integrity and function of the lungs during the development of ALI (11, 12). Two major cell types populate the alveolar epithelium, alveolar epithelial cell type I (AECI) and type II (AECII) cells. AECII cells cover 2–5% of the surface area and have many known functions, including the secretion of surfactant (13, 14). AECI cells constitute the vast majority of the internal surface area (approximately 95%) of the lung, and interact with noxious stimuli during the development of ALI (13, 14). Recent evidence suggests that ATI cells have important functions in innate immunity and are underappreciated players in lung cell–cell crosstalk (15).

### Alveolar Macrophage Polarization, Activation, and Function in the Pathogenesis of Lung Injury

As a first-line defender, AMs are armed with high levels of pathogen-associated molecular pattern and danger-associated molecular pattern receptors in order to initiate necessary immune response (16). In response to the stimulation of microenvironmental signals, AMs often display the M1 macrophage phenotype (classically activated macrophage) or M2 phenotype (alternatively activated macrophage). M1-activated AMs produce high levels of proinflammatory cytokines, including IL-1β, tumor necrosis factor (TNF)-α, IL-12, and iNOS in the presence of IFN-γ or IFN-β (17). M2 macrophages produce anti-inflammatory cytokines, IL-10 and IL-1ra in response to IL-4 and IL-13. M1-activated macrophages often express MHC II (IA/IE), CD80, CD86, and CCR2, while M2-macrophages express mannose receptor, dectin-1, TfR (transferrin receptor), and CD200R (17).

During the development of ALI in animals, AMs are thought to play essential roles in both the acute inflammatory phase and resolution phase. Macrophage-derived cytokines are viewed as the major mediators. Resident AMs generate IL-8 and TNF-α, and subsequently stimulate neighboring cells to propagate the inflammatory responses (18). Increased BAL IL-8 level and increased IL-8 expression in AMs are associated with increased mortality in ARDS patients (18, 19). Additionally, macrophages secrete epithelial growth factor and GM-CSF to promote epithelial repair (20), an example of macrophage–epithelial communications. In order to achieve classical activation (M1) or alternative activation (M2), macrophages constantly receive signals from surrounding or other distant cells. A crosstalk has been reported between AMs and epithelial cells, in particular AECII cells, primarily *via* an autocrine and/or paracrine manner (21). The paracrine communication network between AMs and epithelial cells has been reported to affect alveolar fluid clearance in influenza virus-induced lung injury (22), *via* epithelial type I IFN and especially the IFN-dependent, macrophage-expressed TNF-related apoptosis-inducing ligand (TRAIL). TRAIL determines Na, K-ATPase plasma membrane protein abundance and, thus, edema clearance during IAV infection (22). Appropriate modulation of the epithelial–macrophage crosstalk might represent a novel strategy to improve the unchecked balance of lung inflammation, epithelial damage, and fluid absorption, thus alter the outcomes in lung injury. However, the trials of cytokine suppression or antibody administration have not resulted in any favorable outcomes (23), suggesting unrecognized mechanisms that remain to be explored. For example, the minimum amount of cytokine required to maintain a concentration in the lung microenvironment, the mechanism by which the released cytokines are guided to their target cells. And how are the signaling molecules, including cytokines, proteins, and DNA/RNAs protected from degradation or inactivation by extracellular enzymes?

Recently, emerging evidence suggest that EVs provide further understandings in addition to what we have known on the macrophage–epithelial crosstalk *via* cytokines and chemokines.

### EVs: Newly Recognized “Organelles”

**Figure 1:**
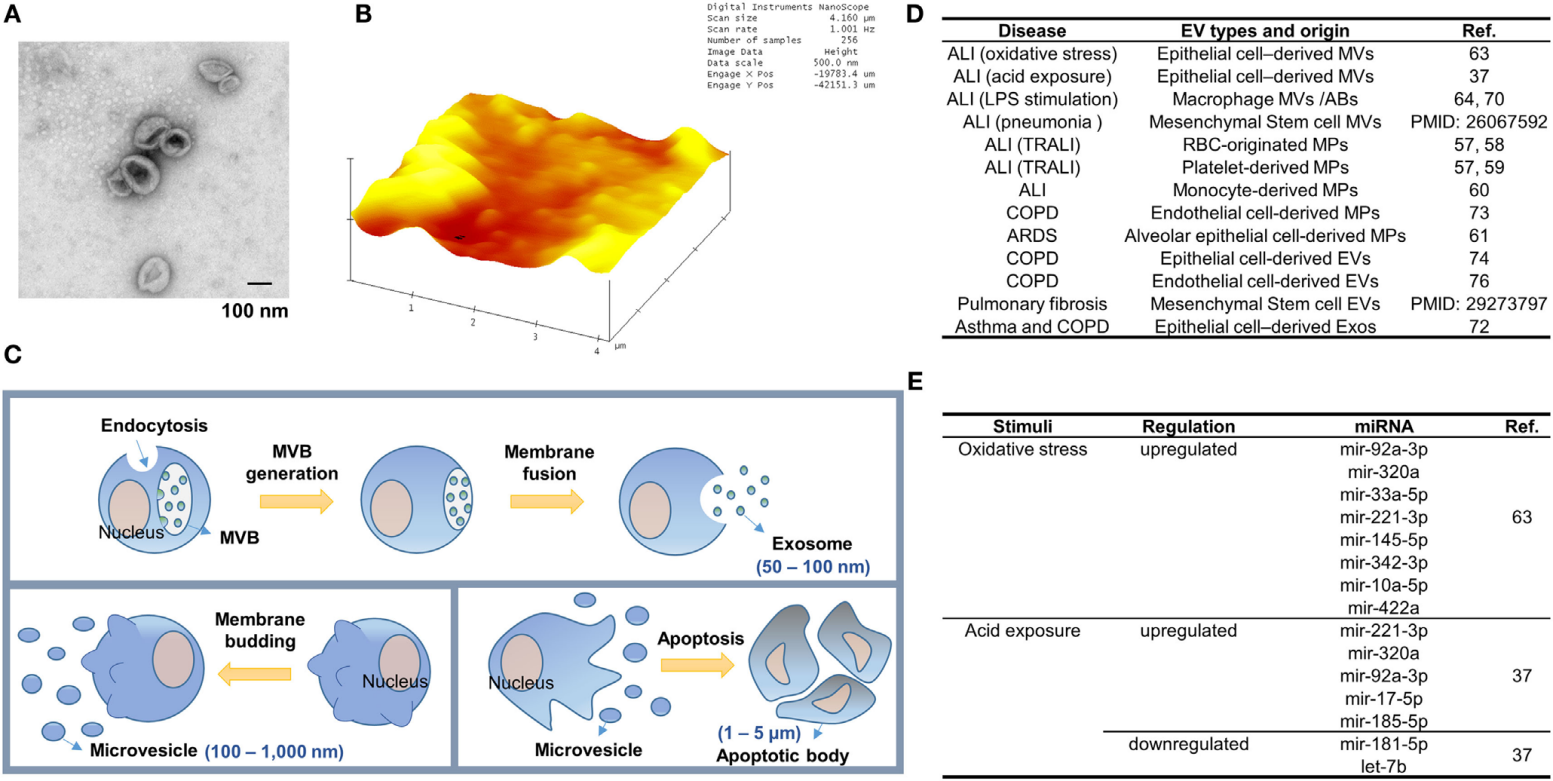
Extracellular vesicle (EV) generation during lung inflammation. **(A,B)** Transmission electron microscopy (TEM)-based **(A)** and atomic force microscopy (AFM)-based **(B)** structure images of EVs derived from bronchoalveolar lavage fluid. EVs were fixed and dried on the formvar coated TEM grids (Ted Pella, Redding, CA, USA) and the cleaved mica sheets (Grade V-1, thickness 0.15 mm, size 15 × 15 mm) for TEM and AFM analysis, respectively. **(C)** Three main types of EVs. Exosome generation is initiated by membrane-endocytosis and inward-budding of the endosomal membranes to form multiple vesicular bodies (MVBs). Exosomes are then released when the MVBs are fused with the plasma membrane of the cells. Meanwhile, microvesicles (MVs) are formed by outward-budding of the plasma membrane. The size of MVs (100 nm–1 µm) is bigger than exosomes (50–100 nm) and their production is stimulated in various cell-stress conditions. Apoptotic bodies (ABs) are formed by membrane-blebbing of apoptotic cells. ABs are the largest EVs (1–5 µm) and contain nuclear fragments. **(D)** The type of EVs released during the development of lung inflammation and injury. **(E)** Epithelial MV-containing miRNAs altered in sterile ALIs.

**Figure 2:**
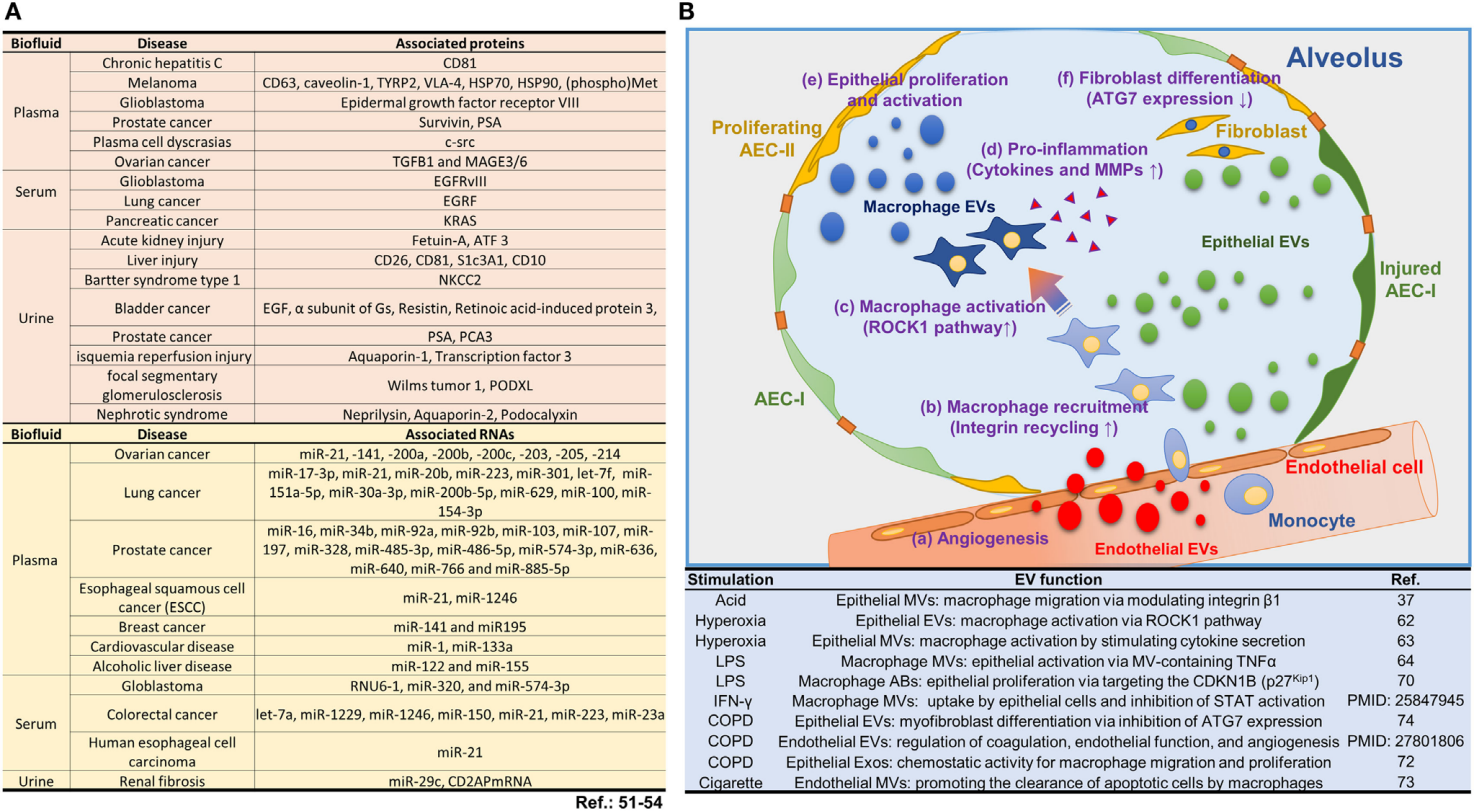
Roles of extracellular vesicles (EVs) in lung injury. **(A)** EV-containing molecules reported to potentially serve as biomarkers. **(B)** The current reports on EVs and their functions involved in lung injury and inflammation. Schematic illustration and the summarized table for the biological pathways by which EVs contribute to the alveolar inflammatory process.

Extracellular vesicle-like molecules were initially described by Chargaff and West in 1946 (24). Currently, EVs have been isolated from almost all cell types and biological fluids, including broncho-alveolar lavage fluid (BALF). The morphology and structure of EVs can be visualized under transmission electron microscopy (Figure 1A) and 2D view (Figure 1B). In the past decade, accumulating evidence suggests that EVs play a crucial role in intercellular communication and inter-organ crosstalk.

## Classifications, Nomenclature, and Biogenesis of EVs

According to the International Society of EVs, three main subgroups of EVs have been classified based on the size of EVs, the membrane compositions, and the mechanisms of formation (25). As illustrated in Figure 1C, apoptotic bodies (ABs) are the largest EVs and formed in the process of undergoing apoptosis. Microvesicles (MVs) are the second subgroup measuring approximately 200–500 nm in diameter, comprising of different sized vesicles directly protruding from plasma membranes. Exosomes are the smallest subgroup among EVs measuring approximately 30–100 nm, and are released after multiple vesicular bodies (MVBs) fuse with the plasma membrane [Figure 1C; (26)]. The mechanisms of formation of EVs are also heterogeneous. ABs are generated by cell membrane-blebbing resulting from systematic cellular breakdown during the process of apoptosis. The generation of exosomes is tightly associated with the dynamic homeostasis of endosomes/lysosomes, trans-Golgi network, the MVBs, and intra luminal vesicles. ESCRT machinery plays an essential role in the formation of polymeric filaments and subsequently results in ILV formation, once released, called exosomes. ESCRT protein components have been confirmed in exosomes. Furthermore, ESCRT-independent mechanisms involving ceramide and tetraspanin CD63 have been reported in the exosome biogenesis and release (27–29). Fusion machinery, such as the SNARE proteins and GTPases, has been shown to regulate the ILV-plasma membrane fusion. Examples of such machineries are SNARE proteins and Rab GTPases (30, 31). As shown in Figure 1C, MVs are formed *via* the outward budding and expulsion of plasma membrane directly from the cell surface. This process of vesicle formation is often triggered by translocation of phosphatidylserine to the outer-membrane leaflet through aminophospholipid translocase activity (32, 33). MV formation is an energy-consuming process and requires ATP (34, 35).

## EV Compositions

Microvesicles and exosomes are highly enriched with a variety of components and surface marker. A subset of marker proteins derived from parent cells is often detectable in EVs. Surfactant proteins, marker of alveolar type II cells (AECII), and caveolin-1, marker of alveolar type I cells (AECI) can be detected in the EVs derived from lung epithelial cells (34–37). MVs and exosomes also carry distinct proteins which can be used to differentiate the two types of EVs. Vesicle-associated membrane protein 3 can be found in the MVs while transferrin receptors are highly enriched in exosomes, but not in the MVs (38, 39). The marker proteins of MVs or exosomes are related to the parent cells and mechanism of secretion, thus can be used to distinguish the types of EVs, i.e., MVs vs exosomes vs ABs, as well as their origins. EV-encapsulated cytokines are a group of key proteins which potentially transmit inflammatory signals among cells. Examples of the EV-carrying cytokines include but not limit to interleukin 1β (IL-1β), IL1α, IL-18, macrophage migration inhibitory factor, IL-32, TNF, IL-6, vascular endothelial growth factor, IL-8 (CXCL8), fractalkine (CX3CL1), CCL2, CCL3, CCL4, CCL5, and CCL20 (40). Identifications of these important immune-modulatory cytokines/chemokines in EVs strongly indicate that EVs carry crucial cellular functions and mediate intercellular communication.

RNAs detected in EVs generally are much smaller than cellular RNAs [less than 700 nucleotides (nt)]. Despite the smaller sizes of EV-RNAs, long non-coding RNAs, Ribosomal RNA, and the fragments of these intact RNA molecules have all been found in EVs (26, 41, 42). A large amount of 3′UTR mRNA fragments have been identified in EVs (43). There are multiple microRNA (miRNA) binding sites on the 3′UTR mRNAs (44) and a variety of miRNAs have been identified in EVs, suggesting that EVs serve as a cargo for circulating miRNAs. However, MVs appear to be the main cargo carrying majority of miRNAs, recent studies have highlighted that there are various number of copies of “highly up-regulated” miRNAs found in tumor cells, and very low exosome detected in plasma (45).

## EV Functions and Their Significance

Current understanding on EVs facilitates to fill the knowledge gap on cell–cell communications. For example, EVs may partially answer the questions on how cytokines/chemokines achieve the needed concentration in the microenvironment and reach their target cells. It has been reported that cytokines are not transmitted in free forms, but appear to be associated with EVs (46). Cytokines, chemokines, protein, and miRNAs are markedly enriched inside EVs, suggesting that EV function as a vehicle to concentrate and transport these signaling molecules. Additionally, EV-encapsulating RNAs are protected from RNaseA, and thus EVs provide a consistent source of miRNAs for therapeutic delivery and disease biomarker detection (47). EV-based drug delivery offers several advantages over conventional drug delivery systems: EVs exhibit increased stability in the blood that allows them to travel long distances within the body under both physiological and pathological conditions (48, 49). For example, EVs in plasma are stable up to 90 days under various storage conditions (50). In contrast, peak concentrations of TNF-alpha occur approximately 2 h after administration followed by a rapid decline of free TNF-alpha concentration in plasma (half-life approximately 18.2 min) (5). Moreover, EVs express the same surface markers as their “mother” cells. This feature potentially provides an opportunity to deliver EV-containing molecules in a cell type-specific manner. Furthermore, EVs carrying cell type-specific markers may serve as a diagnostic agent referring as “liquid biopsy” to avoid invasive tissue diagnosis. In fact, many EV-containing molecules have been reported to potentially serve as biomarkers as shown in Figure 2A (51–54).

### MVs Mediate the Intercellular Crosstalk

#### The Type of EVs Released During the Development of Lung Inflammation and Injury

Extracellular vesicle-mediated signal transfer among lung cells is increasingly recognized as a novel mechanism by which the innate immune response is initiated (Figure 1D). A decade ago, scattered reports have shown the association between ALI and the generation of “microparticles” (MPs) derived from platelets, neutrophils, monocytes, lymphocytes, red blood cells, and endothelial and epithelial cells (55, 56). The MPs are now believed to be replaced with the term of MVs.

Initial observations on the potential roles of MPs were made in the transfusion-associated acute lung injury (TRALI) (57). Stored packed RBCs release MPs and these RBC-originated MPs contribute to neutrophil priming, activation, and transfusion-associated ALI (TRALI) (57, 58). Platelet-derived microparticles (PMPs) which carry the sCD40L increase during the storage period. PMPs may contribute to the occurrence of TRALI (57, 59). Furthermore, the signal transmission from monocyte/macrophage to epithelial cells has also been identified. Monocyte-derived MPs upregulate the synthesis of pro-inflammatory factors in lung epithelial cells *via* NF-κB activation through a PPAR-γ-dependent pathway (60).

Alveolar epithelial cell-derived “MPs” have been reported to be the main source of tissue factor procoagulant activity in ARDS (61). EVs detected in BALF may be derived from multiple different cell types, including but not limited to alveolar and bronchial epithelial cells, endothelial cells, AMs, neutrophils, lymphocytes fibroblasts, and the above-mentioned blood cells. The type of EVs detected in BALF, i.e., MVs, exosomes, or ABs, may be dependent on the type of noxious stimuli and the severity of diseases in a time/dose-dependent dynamic manner. Moon and Lee et al. recently described that MVs form the dominant type of EVs in BALF after exposure to oxidative stress (62, 63). Lee et al. further reported that MVs are also the main type of EVs detected in BALF after exposure to acid inhalation (37). Approximately, 70% of BALF-EVs are MVs based on the size and marker analysis, using Nano Tracking Analysis (NTA) and Western Blot Analysis, respectively (37, 63). In the setting of hyperoxia or acid exposure, the second largest group of EVs is composed of exosomes, followed by a small amount of ABs. Apparently, Moon and Lee et al. focused on the early stage of exposure to noxious stimuli, whether the MVs remain dominant type of EVs after prolonged exposure to oxidative stress or acid requires further investigation. Interestingly, in LPS-induced lung injury model, a fair amount of large size vesicles are detected in BALF (64). Since the size of EVs in this study is not analyzed using NTA, the current “state of the art” analysis used in EV research, one potentially argues that the EVs studied in this report are composed of AB and MV mixture.

#### The Source of MVs Detected in BALF During the Development of Lung Inflammation and Injury

Moon et al. also report that at the usual state of mice, i.e., without exposure to noxious stimuli, AMs are the main sources of the MVs detected in BALF (62) (Figure 1D). After exposure to hyperoxia-associated oxidative stress, MVs derived from alveolar epithelial cells increase robustly in BALF. On the other hand, AM-derived MVs remain at a steady level (62). MVs derived from other cells failed to increase as robust as the epithelial cell-derived MVs (62). Lee et al. further confirmed this observation in the setting of acid-exposure induced lung injury (37). In both studies, the type of EVs was analyzed using FACS analysis *via* a bead-based antibody conjugation against the surface markers of interested cells, such as AMs, AECI, or AECII cells.

These studies used non-infectious or sterile stimuli (hyperoxia or acid inhalation). Alveolar epithelium has a large surface area which is exposing to the inhaled stimulators. Hyperoxia-induced oxidative stress and acid inhalation are both known to cause diffuse alveolar cell damage (65–69). Therefore, it is expected that majority of MVs in BALF are derived from lung epithelial cells. On the other hand, bacterial infection often triggers extensive pro-inflammatory responses to induce bactericidal effects. Presumably, after inhaled bacteria or LPS, the first responder which is AM may be responsible for the release of EVs into BALF. Furthermore, Moon and Lee et al. focus on the MV release in BALF at the early stage of exposure rather than prolonged treatment. All the above noxious stimuli, including both sterile and the infectious, potentially induce the generation of ABs after prolonged exposure. Zhu et al. reported recently that AM-derived ABs exert a functional role on the epithelial cells and potentially promote epithelium proliferation (70). Their work confirmed that there is a mutual communication between epithelial cells and AMs, rather than a single direction crosstalk.

### The Compositions of MVs During the Development of Lung Inflammation and Injury

Microvesicles are highly enriched with proteins, lipids, DNA, and RNA molecules (63, 71). Lee et al. first determine the amount of protein and RNAs in the isolated MVs. Although both components are highly upregulated in the presence of noxious stimuli, only RNA components are robustly increased in each individual MV after normalization with the number of MVs. Furthermore, Lee et al. demonstrated that small RNA molecules are elevated much more significantly than the large RNAs (63). Subsequent miRNA profiles and RT-PCR confirmation suggest that after oxidative stress, epithelial MV-containing miRNAs are dramatically altered (Figure 1E).

Functionally, Lee et al. showed that MV-miRNAs promote macrophage migration and infiltration *in vitro* and *in vivo*. After exposure to acid, epithelial MV-containing miR-17 and miR-221 exert the effects on promoting macrophage migration *via* modulating integrin β1 (37). After exposure to hyperoxia, MV-containing miR-221 and miR-320 activate AMs by stimulating pro-inflammatory cytokine secretion (63). It appears that after specific stimuli, different MV-containing miRNAs exert specific functional roles. Collectively, in response to sterile stimuli such as hyperoxia or acid inhalation, AMs receive “pro-inflammatory” signals from the epithelial MV-containing miRNAs and subsequently respond by classical activation (M1) and increased migration.

### Macrophage-Derived EV-Containing miRNAs Regulate Lung Epithelial Cell Proliferation and Cell Cycle

The EV research focused on the roles of MVs or Exos, Zhu et al. recently demonstrate that after LPS stimulation, ABs derived from macrophages exert a functional role in maintaining lung epithelial cell growth *via* their regulation of cell cycle (70). Although AB is significantly larger in size and contains more diverse contents than MV and exosome, Zhu et al. demonstrated that AB-containing miR-221/222 confer robust effects on promoting epithelial cell proliferation *via* targeting the CDKN1B (p27^Kip1^) gene (70). This observation demonstrates that under certain condition, an EV-mediated macrophage-epithelium crosstalk exists in both directions, further confirming a constant and dynamic intercellular communication among different cell types in the microenvironment of lungs.

### EVs Play a Role in Other Inflammatory Lung Responses

Apart from ALI and inflammation, the generation and function of EVs in the pathogenesis of other lung disease have gained increasing attention. For example, in the development of COPD, EV-mediated signaling transport has been widely reported (72– 74). Epithelial cell-derived exosomes have been detected from BALF of control and asthmatic mice (72), in response to IL-13. These epithelial exosomes induce chemotaxis of undifferentiated macrophages and confer proliferative effects (72). Despite that in this report, due to the lack of NTA analysis of the sizes of EVs, the term “exosome” here may represent the three groups of EVs.

Cigarette smoke has been reported to induce the endothelial cell-derived MVs and MV-containing miRNAs, such as miR-191, miR-126, and miR125a. These miRNAs are transferred to macrophages in an EV-mediated manner, subsequently promoting the clearance of apoptotic cells (73). Interestingly, besides AMs, lung epithelial EVs can also transport EV-containing miRNA, miR-210, into lung fibroblasts, resulting in the inhibition of ATG7 expression and promotion of myofibroblast differentiation (74).

In addition to the EVs derived from lung epithelial cells, endothelial cells also generate a significant amount of EVs. Takahashi et al. have demonstrated that endothelial cell-derived MVs increase robustly in COPD patients compared to those in healthy volunteers (75). Furthermore, the injured endothelial cells release a significant amount of EVs, which regulate the process of coagulation, inflammation, endothelial function, and angiogenesis (76). The current reports on EVs and their functions involved in lung injury and inflammation are summarized in Figure 2B.

### Pitfalls and Further Directions

Many questions remain to be answered on the role of EVs in the cell–cell crosstalk during the development of lung inflammation and injury. These questions include but are not limited to the concentration and amount of specific miRNAs in each MVs, exosomes or ABs after noxious stimuli; the effective “dose” or “amount” of MV/AB-shuttling miRNAs to trigger cellular effects; the efficacy and pathway of MV or AB-shuttling miRNAs to enter the recipient cells. There is yet to be a study on the underlying mechanisms by which EV-shuttling miRNAs exert functions in the recipient cells.

In summary, EVs (MVs, exosomes, or ABs) play an essential role in mediating epithelial–macrophage crosstalk in the absence and presence of noxious stimuli. EV-containing miRNAs are the likely emerging targets for the development of novel therapeutic and/or diagnostic agents.

## Author Contributions

YJ designed, wrote, and supervised this manuscript. HL and EA wrote the manuscript, drew the schema. DZ and AR participated in the writing of the manuscript. HL and EA contributed equally to this work.

## Conflict of Interest Statement

The authors declare that the research was conducted in the absence of any commercial or financial relationships that could be construed as a potential conflict of interest.
